# Seroprevalence of flaviviruses antibodies in water buffaloes (*Bubalus bubalis*) in Brazilian Amazon

**DOI:** 10.1186/1678-9199-20-9

**Published:** 2014-03-25

**Authors:** Alexandre R Casseb, Andrea V Cruz, Iroleide S Jesus, Jannifer O Chiang, Lívia C Martins, Sandro P Silva, Daniele F Henriques, Livia MN Casseb, Pedro Fernando C Vasconcelos

**Affiliations:** 1 Institute of Health and Livestock Production, Federal Rural University of the Amazon (UFRA), Belém, Pará State, Brazil; 2 Department of Arbovirology and Hemorrhagic Fevers, Evandro Chagas Institute, Ananindeua, Pará State, Brazil; 3 Institute of Biological Sciences, Federal University of Pará (UFPA), Belém, Pará State, Brazil; 4 Department of Pathology, State University of Pará (UEPA), Belém, Pará State, Brazil; 5 Instituto da Saúde e Produção Animal, Universidade Federal Rural da Amazônia, Av. Presidente Tancredo Neves, 2501, Belém, PA, 66077-901, Brazil

**Keywords:** *Flavivirus*, Water buffaloes, Hemagglutination inhibition test

## Abstract

**Background:**

The state of Pará encompasses 26% of Brazilian Amazon where an enormous diversity of arboviruses has been found. This study aimed to assess the prevalence and distribution of hemagglutination-inhibition antibodies against antigens of six *Flavivirus* (yellow fever virus, Ilheus virus, Saint Louis encephalitis virus, Cacipacore virus, Bussuquara virus and Rocio virus) in water buffaloes in Pará state, Brazil. The prevalence of antibodies in these farm animals is important to determine the circulating arboviruses.

**Findings:**

All investigated arboviruses were detected in the species studied and our results indicate that water buffaloes are susceptible to *Flavivirus* infection. Furthermore, there is solid evidence of active circulation of these viruses in the Brazilian Amazon.

**Conclusions:**

Water buffaloes showed higher prevalence of heterotypic antibody reactions and we hypothesized that they can serve as sentinels to detect the movement of such arboviruses in the Brazilian Amazon.

## Findings

Nearly 26% of the Brazilian Amazon belongs to the Pará state and it is estimated that the buffalo herd of this region represents approximately 45% of the Brazililian population, found mainly on the Marajó island [1]. With few exceptions, arbovirus (arthropod-borne virus) infections comprise zoonosis, because their natural cycles involve non-human vertebrates (hosts) and hematophagous arthropods (vectors). The Pan-Amazon is the largest area in the world where those viruses are found, and the Brazilian Amazon alone houses the largest variety of known isolated arboviruses [2].

In the Brazilian Amazon, there are numerous species of hematophagous diptera and wild vertebrates that cohabit within the same ecosystem. Such diversity and increased population size is globally unique. These factors provide favorable environmental conditions for viruses, particularly arboviruses [3].

Although 210 arboviruses have been isolated in Brazil, their vast majority have been found in the Brazilian Amazon [4,5]. To the best of our knowledge, there have been no studies focused on investigating the role of water buffaloes (*Bubalus bubalis*) in the maintenance of arboviruses. To address this question, we have evaluated the prevalence of antibodies in water buffaloes in Pará state in order to determine which arboviruses are circulating.

Blood samples were collected from animals living in the six mesoregions of Pará, which were two years old or more, without arbovirus vaccination and born and raised at the studied areas. After proper restraint of the animals and local asepsis, the jugular vein was punctured, without anticoagulant, with a vacuum system and serum samples were transported on ice and then stored at −70°C until use. A total of 654 serum samples from water buffaloes were collected.

The hemagglutination-inhibition (HI) test was performed according to the microplate-adjusted protocol described by Shope [6]. The test is based on the ability of arboviruses to agglutinate goose (*Anser anser*) red blood cells. It is possible to inhibit hemagglutination activity of antigens using serum containing specific antibodies.

The test was performed against antigens from arboviruses isolated in Brazil of six different *Flavivirus* types: yellow fever virus (YFV), Ilheus virus (ILHV), Saint Louis encephalitis virus (SLEV), Cacipacore virus (CPCV), Bussuquara virus (BSQV) and Rocio virus (ROCV). All these viruses belonged to the collection of the Department of Arbovirology and Hemorrhagic Fevers of Evandro Chagas Institute, Ananindeua, PA, Brazil. Antigens were prepared from infected tissues from brain, liver or serum of newborn mice using the sucrose-acetone extraction technique. Animal sera were tested (initial dilution of 1:20) against four antigen units [7].

Rodrigues *et al.*[8] criteria for positivity were chosen. Positive reactions were classified as monotypic (reaction against only one antigen or the reaction was at least four-fold stronger against one antigen compared to all others of the same viral genus) or heterotypic (reactions against two or more viruses exhibiting similar titers or the predominance of an antigen was less than four-fold when compared to the others) or total (monotypic reactions plus heterotypic reactions).The results were analyzed using the chi-square goodness-of-fit test, using the BioEstat 5.0 software [9]. The sample scores were measured using a significance level (α) of 0.05 to reject the null hypothesis (*p* ≤ α).

Antibodies against the six investigated arboviruses showed different positive reaction rates (Table 1). Positive reactions against specific virus antigens suggest that different flaviviruses circulate among water buffaloes in Pará, Brazilian Amazon. These findings are surprising because there is little published evidence regarding arbovirus infections on buffaloes. In addition, water buffaloes usually live in flooded regions where arthropods are frequently found, thereby increasing the probability of an infection.

**Table 1:** Prevalence of hemagglutination-inhibition antibodies against antigens of six flaviviruses in serum samples of water buffaloes from different regions of Pará state, Brazilian Amazon

**Water Buffaloes (n = 654)**						
**Virus**	**HR**	**%HR**	**MR**	**%MR**	**TR**	**%TR**
SLEV	65	9.94	82	12.54	147	22.48
ILHV	48	7.34	25	3.82	73	11.16
YFV	14	2.14	9	1.37	23	3.51
ROCV	11	1.68	2	0.30	13	1.99
BSQV	2	0.30	4	0.61	6	0.92
CPCV	8	1.22	0	0	8	1.22

HR: heterotypic reactions, MR: monotypic reactions, TR: total reactions, SLEV: Saint Louis encephalitis virus, ILHV: Ilheus virus, YFV: yellow fever virus, ROCV: Rocio virus, BSQV: Bussuquara virus, CPCV: Cacipacore virus.

The present results indicated that SLEV was the arbovirus with the highest HI antibody prevalence, which had a broad circulation compared to other investigated *Flaviviruses* (*p* < 0.05), except for ILHV (*p =* 0.1275). In Brazil, several SLEV strains have been isolated in the neighborhood of Belém, Pará state, from sentinel animals and also during epizootics from both wild birds and hematophagous arthropods over different years and geographical areas [10]. Several serologic surveys previously performed in humans living in the Brazilian Amazon indicated the prevalence of HI antibodies against SLEV ranging from 1 to 5% [11]. In contrast to the human data, the results of the current study indicate that SLEV was the most prevalent virus in all investigated areas. Indeed, a remarkable prevalence of SLEV infection was observed in the studied water buffaloes.

ILHV is the most widely distributed non-epidemic *Flavivirus* and exhibits the highest prevalence by HI assays in Brazil. In the Brazilian Amazon, this virus has been systematically isolated from several wild animal species and vector arthropods, found sporadically from febrile patients [11]. It exhibited the second highest prevalence in this study.

The present study has also detected HI antibodies against YFV in water buffaloes. This virus had the third highest prevalence. Such results are puzzling and may indicate cross-reactivity with another untested or unknown *Flavivirus*, or, alternatively, may indicate that animals are becoming asymptomatically infected by YFV. Further studies must be carried out to establish the prevalence of neutralizing YFV antibodies in these animals.

There are no records of association of the flaviviruses ROCV, BSQV and CPCV with disease in domestic or wild animals [11]. However, some human patients from the state of São Paulo, Brazil, reported domestic swine and fowl deaths during a ROCV encephalitis epidemic in humans [12]. The CPCV had no monotypic reactions that indicate cross-reactivity with other tested *Flavivirus*.

## Conclusions

The current findings allow us to conclude that there is preliminary evidence of active *Flavivirus* infection in water buffaloes in the Brazilian Amazon. In this region, the animals are exposed to many thousands of mosquito bites in places that sometimes serve as transmission foci for these viruses. Animals bitten by infected mosquitoes may develop infection and seroconversion. If frequently exposed to arboviruses, the buffaloes possibly represent a public health risk to humans that may be susceptible to develop the infection. In many cases, high seroprevalence in farm animals indicate lower risk for people, since the animals are providing zooprophylaxis by absorbing infectious bites from mosquitoes. The water buffaloes showed higher prevalence of heterotypic antibodies reactions, which suggests that they might to act as sentinel to detect the movement of arboviruses.

### Ethics Committee Approval

The present study was approved by the Animal Research Ethics Committee (CEPAN) of the Evandro Chagas Institute (IEC) (protocol n. 054/2009 CEPAN/IEC). All procedures involving newborn Swiss albino mice (2–3 days old) and water buffaloes were conducted with utmost care to avoid undue suffering.

## Competing interests

The authors declare that there are no competing interests.

## Authors’ contributions

ARC conducted the sample collection, serological tests and helped write the manuscript; LMNC carried out serological tests and statistical analysis; SPS, DFH, JOC and LCM also performed serological tests for the research; ISJ and AVC took part in sample collection; PFCV wrote and reviewed the manuscript. All authors read and approved the final manuscript.
